# Deficiency of NOX1 or NOX4 Prevents Liver Inflammation and Fibrosis in Mice through Inhibition of Hepatic Stellate Cell Activation

**DOI:** 10.1371/journal.pone.0129743

**Published:** 2015-07-29

**Authors:** Tian Lan, Tatiana Kisseleva, David A. Brenner

**Affiliations:** 1 Department of Medicine, University of California San Diego, La Jolla, California, United States of America; 2 Vascular Biology Research Institute, Guangdong Pharmaceutical University, Guangzhou, China; University of Navarra School of Medicine and Center for Applied Medical Research (CIMA), SPAIN

## Abstract

Reactive oxygen species (ROS) produced by nicotinamide adenine dinucleotide phosphate oxidase (NOX) play a key role in liver injury and fibrosis. Previous studies demonstrated that GKT137831, a dual NOX1/4 inhibitor, attenuated liver fibrosis in mice as well as pro-fibrotic genes in hepatic stellate cells (HSCs) as well as hepatocyte apoptosis. The effect of NOX1 and NOX4 deficiency in liver fibrosis is unclear, and has never been directly compared. HSCs are the primary myofibroblasts in the pathogenesis of liver fibrosis. Therefore, we aimed to determine the role of NOX1 and NOX4 in liver fibrosis, and investigated whether NOX1 and NOX4 signaling mediates liver fibrosis by regulating HSC activation. Mice were treated with carbon tetrachloride (CCl_4_) to induce liver fibrosis. Deficiency of either NOX1 or NOX4 attenuates liver injury, inflammation, and fibrosis after CCl_4_ compared to wild-type mice. NOX1 or NOX4 deficiency reduced lipid peroxidation and ROS production in mice with liver fibrosis. NOX1 and NOX4 deficiency are approximately equally effective in preventing liver injury in the mice. The NOX1/4 dual inhibitor GKT137831 suppressed ROS production as well as inflammatory and proliferative genes induced by lipopolysaccharide (LPS), platelet-derived growth factor (PDGF), or sonic hedgehog (Shh) in primary mouse HSCs. Furthermore, the mRNAs of proliferative and pro-fibrotic genes were downregulated in NOX1 and NOX4 knock-out activated HSCs (cultured on plastic for 5 days). Finally, NOX1 and NOX4 protein levels were increased in human livers with cirrhosis compared with normal controls. Thus, NOX1 and NOX4 signaling mediates the pathogenesis of liver fibrosis, including the direct activation of HSC.

## Introduction

Liver fibrosis occurs as a result of chronic liver disease and is associated with severe morbidity and mortality [1]. Chronic oxidative stress is an important etiological factor in initiating the fibrogenic process in the liver [2]. Hepatic stellate cells (HSCs) are endogenous, liver-specific mesenchymal cells that play pivotal roles in liver inflammation and fibrogenesis [1]. In the normal liver, HSCs are quiescent, desmin-positive cells, containing vitamin A lipid droplets. Upon activation by liver injury, quiescent HSCs become activated HSCs, characterized by expression of α-smooth muscle actin (α-SMA) [3], producing inflammatory cytokines, chemokines and extracellular matrix proteins [4] [5].

Reactive oxygen species (ROS) are generated by various liver injuries such as alcohol abuse, hepatitis virus infection and chronic cholestasis and contribute to hepatic fibrogenesis [6]. ROS stimulates the production of the Collagen I, acting as an intracellular signaling mediator of the fibrogenic action of TGF-β1 [7]. The multicomponent nicotinamide adenine dinucleotide phosphate (NADPH) oxidase (NOX) enzyme complexes and the mitochondrial respiratory pathway are the two major producers of endogenous ROS [8]. NOX play a central role in liver fibrogenesis. Among the seven members of the NOX family, NOX1 is structurally and functionally similar to NOX2, the classic NOX that generates the oxidative burst in neutrophil killing. Studies by us and others have shown that NOX1 and NOX2 are expressed in HSCs and deficiencies of NOX1 or NOX2 decrease liver inflammation and fibrosis in the carbon tetrachloride (CCL4) and bile duct ligation (BDL) models [5, 9]. Angiotensin II (Ang II) also induces NOX1 to promote HSCs proliferation and aggravate liver fibrosis [5, 9]. In contrast, NOX4, a nonphagocytic NOX homolog is expressed in the liver, and is different from the other NOX isoforms because it does not require the recruitment of cytosolic structural subunits to form the active enzyme to produce ROS [10, 11]. NOX4 is critical in lung and kidney fibrosis by activating and transforming of myofibroblasts [12, 13]. In the liver, NOX4 is expressed in hepatocytes, stellate cells, and endothelial cells [14]. NOX4 has been found to be upregulated in hepatitis virus C, and to contribute to the formation of ROS, most likely via TGF-β1 induction [10]. The role of NOX4 in liver injury and fibrosis has only been assessed in the BDL model using NOX4 deficient mice [15]. A concern about these previous studies is that they were performed by breeding homozygous knock-out mice compared to wild type strain matched control mice, which could result in artifact genetic drift in the two groups.

Recently, small molecule NOX1/4 dual inhibitors such as GKT137831 have been developed that show good orally bioavailability and tolerability when administered orally in animal model of pulmonary fibrosis [16] and liver fibrosis [15]. Thus, we hypothesize that deficiency of either NOX1 or NOX4 attenuates HSCs activation and liver fibrosis.

The overall goal of this study was to determine the roles of NOX1 and NOX4 on the proliferative and fibrogenic phenotypes of HSCs and its contribution to liver fibrosis. We report for the first time a direct comparison of the long-term effects of NOX1 and NOX4 deficiency in the development and progression of liver fibrosis, by comparing liver fibrosis in CCl_4_-induced NOX1KO and NOX4KO mice and their respective wild-type (WT) littermates. Our results demonstrate that both NOX1 and NOX4 play important roles in liver fibrosis in HSCs, and that NOX4 has a more robust role in the activation of HSCs.

## Materials and Methods

### Chemical and Reagents

Ang II, Lipopolysaccharides (LPS), Platelet-derived growth factor (PDGF) were purchased from Sigma-Aldrich (St. Louis, MO). Murine recombinant Shh was obtained from R&D Systems, USA). 2’7’-dichlorofluorescein diacetate (CM-H_2_DCFDA) was purchased from Molecular Probe Inc. (Eugene, OR). Enhanced luminescence system for superoxide detection (Diogenes) was purchased from the National Diagnostics (Atlanta, GA). An OxiSelect TBARS assay kit for MDA quantification was purchased from Cell Biolabs (San Diego, CA). GKT137831. 2-(2-chlorophenyl)-4-[3-(dimethylamino)phenyl]-5-methyl-1H-pyrazolo[4,3-c]pyridine-3,6(2H,5H)-dione was provided by Genkyotex S.A. (Geneva, Switzerland). The NOX1 and NOX4 antibodies were purchased from Boster Biological Technology Company, Wuhan, China.

### Mice

Mice with deletions in the Nox1 and Nox4 genes were generated by Dr. K.H. Krause as described [17]. Mice heterozygous for either the Nox1 or Nox4 deletion were crossed to generate homozygous Nox1 or homozygous Nox4 knockout mice for experiments. The WT littermates were used as genotype controls and oil injection was used as vehicle controls. The CCl_4_ study was performed as described [15] consisted of WT mice treated with oil (6 mice), NOX1 KO mice treated with oil (6 mice), NOX4 KO mice treated with oil (6 mice), WT mice treated with CCl_4_ (10 mice), NOX1 KO mice treated with CCl_4_ (8 mice), and NOX4 KO mice treated with CCl_4_ (8 mice).

### Ethics Statement on Animal Care

All animal were conducted according to the Institutional Animal Care and Use Committee (IACUC) of University of California, San Diego (UCSD), Animal Use Protocol S07088 (approved 1/17/2013). Veterinary Health Unit of the University of California, San Diego’s Animal Care Program provides the veterinary care and consultation on a 24 hours basis (including weekends and holidays) by a staff of veterinarians with specialties in laboratory animal medicine and anesthesiology, and licensed animal health technicians with veterinarians as a routine protocol and implementation at UCSD. Animal welfare: Animal discomfort is minimized by caging in an approved environment and skillful handling by trained personnel. Mice showing signs of ill health will be monitored closely and euthanized if necessary to limit suffering. Prior to the start of any experiment involving a live animal that involves opening the abdomen of the animal, the animal is anesthetized with a dose of Xylazine and Ketamine given IP. No procedure is commenced until the animal reaches a deep state of unconsciousness, as indicated by the lack of a response to a toe pinch. Euthanasia to be used and the reasons for its selection. Euthanasia is performed by either CO2 or isoflurane anesthesia. Isoflurane is an odorless and painless inhalant that causes the animal no distress. The animals are not allowed to awaken during any procedure. All animals painlessly expire while in a deep state of unconsciousness in a chamber containing an isoflurane-saturated atmosphere. This method was chosen based on the recommendation of UCSD’s Animal Care Program and is consistent with the recommendations of the Panel on Euthanasia of the American Veterinary Medical Association.

### Human Liver Analysis

Liver Samples were taken from the explants of 10 patients with clinically diagnosed liver cirrhosis who underwent liver transplantation. Liver tissues with normal histology (n = 7) obtained from patients with various benign liver conditions or transplant donors were used as controls. All clinical samples are from Nanfang Hospital of the Southern Medical University, Guangzhou, China. Written informed consent was obtained from each patient and volunteer, and the study was approved by the Clinical Ethics Committee of Nanfang Hospital.

### Serum Biochemistry

Serum levels of alanine aminotransferase (ALT) and aspartate aminotransferase (AST) were measured using standard enzymatic procedures according to the manufactures’ instruction (Thermo Fisher, Pittsburgh, PA, USA).

### Assessment of Hepatic Fibrosis

Hepatic fibrosis was assessed by morphometric analysis of the Sirius Red-stained area. For Sirius Red staining, liver tissues were fixed in 10% buffered formalin, embedded in paraffin and sectioned at 5 μm thickness. Sections were stained with Sirius Red solution (saturated picric acid containing 0.1% Direct Red 80) to visualize collagen deposition. The Sirius Red-positive area was measured in low-power (x4 and x10) fields on each slide and quantified using NIH imaging software.

### Thiobarbituric Acid Reactive Substances (TBARS) Determination

Hepatic lipid peroxidation was assessed by TBARS formation. Briefly, liver tissues were homogenized in PBS containing 1X BHT using a polytron homogenizer. One hundred microliters of samples were incubated with 100 μl of SDS lysis solution and 250 μl of 0.52% (pH 3.5) aqueous solution of thiobarbituric acid. After heating at 95°C for 55 minutes, the samples were centrifuged at 3000 rpm for 15 minutes. The red pigment in the supernatant fractions was estimated by spectrophotometric plate reader (Sepctramax M3, Molecular Devices) at 532 nm absorbance. A calibration curve was prepared with an MDA standard. Results were expressed as nmol MDA/mg protein. The results from all of samples were within the linear portion of the MDA standard curve.

### Immunohistochemistry and Immunofluorescence in Mouse Liver Tissue

For immunohistochemical analysis, mouse liver sections were deparaffinized, rehydrated and incubated with anti-desmin antibody (1:200, Thermo Fisher Scientific, Fremont, CA), anti-α-SMA antibody (1:200, Abcam, Cambridge, MA) or anti-F4/80 (1:200, eBioscience, San Diego, CA) and stained using DAKO Envision system (DAKO Corp., Carpinteria, CA). The area of positive staining was measured in low-power (x10) fields on each slide and quantified using NIH imaging software. For immunofluorescent staining, the sections were incubated with anti-HNE (1:200, Alpha Diagnostics, San Antonio, TX), anti-PCNA (1:200, Biolegend Inc., San Diego, CA), anti-desmin (1:200, Thermo Fisher Scientific, Fremont, CA), or anti-Ki67 (1:200, GeneTEX, INC. Irvine, CA) antibodies and Alexa Fluor 594- and Alexa Fluor 488-conjugated secondary antibodies (1:200, Invitrogen, Carlsbad, CA) and imaged with fluorescent microscopy.

### Western Blotting

Electrophoresis of protein extracts and subsequent blotting were performed. Blots were incubated with mouse anti-α-SMA (Abcam, Cambridge, MA) or anti-PCNA (Biolegend Inc., San Diego, CA) at a dilution of 1:1,000. After incubation with HRP-conjugated secondary antibody, blots were visualized by the enhanced chemiluminescence method (Amersham Biosciences). Blots were reprobed with anti-α-tubulin mouse antibody (Santa Cruz Biotechnology) to demonstrate equal loading.

### Cell Isolation and Treatment

Primary HSCs were prepared from mice using a two-step collagenase-pronase perfusion of mouse livers as described previously [9]. After isolation, HSCs were seeded on plastic tissue-culture dishes and cultured in Dulbecco’s modified Eagle’s medium (DMEM) (Gibco, Grand Island, NY), supplemented with 10% fetal bovine serum. HSCs isolated from WT, NOX1KO or NOX4KO mice were culture for 3 days, followed by incubation of Ang II (10^-6^ M), LPS (100 ng/ml), PDGF (10 ng/ml), Shh (1 μg/ml) or vehicle (PBS) with NOX1/4 inhibitor GKT137831 (20 μM) or vehicle (PBS) for 6, 24, or 48 h, individually.

### Measurement of ROS levels in HSCs

Primary HSCs were preincubated with redox-sensitive dye, 2’7-dichlorofluorescein diacetate (DCFDA) (10 μM; Molecular Probes, Eugene, OR) for 20 min, then stimulated with Ang II (10^-6^ M), LPS (100 ng/ml), PDGF (10 ng/ml), Shh (1 μg/ml) or vehicle (PBS) in the presence or absence of GKT137831 (5 μM) for 60 min. DCFDA fluorescence was measured with a multiwall fluorescence scanner.

### Quantitative Real-time RT-PCR

Total RNA was extracted from isolated liver cell fractions and mouse liver tissues by homogenization and purification using TRIzol reagent (Invitrogen, Carlsbad, CA) followed by treatment with RNase-free DNase (Ambion, Austin, TX) for 30 min at 37°C. RNA was reverse-transcribed using a first-strand cDNA kit with random primers (Applied Biosystems, Foster City, CA) according to the manufacturer's protocol. Real-time quantitative PCR of samples was performed for 40 cycles of 15 seconds at 95°C and 60 seconds at 60°C using ABI Prism 7000 Sequence Detector (Applied Biosystems) and SYBR Green mastermix (Applied Biosystems) and primers. The relative abundance of the target genes was obtained by calculating against the standard curve and normalized to HPRT RNA as internal controls. Sequences of PCR primers are summarized in Table 1.

**Table 1:** Primer Sequences for Real-time.

PCR Gene	Accession number	Forward primer (5’-3’)	Reverse primer (5’-3’)
NOX1	NM_172203	TCGACACACAGGAATCAGGA	TTACACGAGAGAAATTCTTGGG
NOX4	NM_015760	TCAGGACAGATGCAGATGCT	CTGGAAAACCTTCCTGCTGT
Collagen α1 (I)	NM_007742	TAGGCCATTGTGTATGCAGC	ACATGTTCAGCTTTGTGGACC
α-SMA	NM_007392	GTTCAGTGGTGCCTCTGTCA	ACTGGGACGACATGGAAAAG
TGFβ1	NM_011577	GTGGAAATCAACGGGATCAG	ACTTCCAACCCAGGTCCTTC
TIMP1	NM_011593	AGGTGGTCTCGTTGATTTCT	GTAAGGCCTGTAGCTGTGCC
PDGF	NM_011057	CCTTCCTCTCTGCTGCTACC	GAAGATCATCAAAGGAGCGG
F4/80	X93328	CTTTGGCTATGGGCTTCCAGTC	GCAAGGAGGACAGAGTTTATCGTG
TNF-α	NM_013693	AGGGTCTGGGCCATAGAACT	CCACCACGCTCTTCTGTCTAC
MCP-1/Ccl2	NM_011333	ATTGGGATCATCTTGCTGGT	CCTGCTGTTCACAGTTGCC
IL-1β	NM_008361	TGCCACCTTTTGACAGTGATG	ATGTGCTGCTGCGAGATTTG
MIP-1/Ccl4	NM_013652	GAAACAGCAGGAAGTGGGAG	CATGAAGCTCTGCGTGTCTG
Cxcl1	NM_008176	TCTCCGTTACTTGGGGACAC	CCACACTCAAGAATGGTCGC
Cxcl2	NM_009140	TCCAGGTCAGTTAGCCTTGC	CGGTCAAAAAGTTTGCCTTG
Ccl3	NM_011337	GTGGAATCTTCCGGCTGTAG	ACCATGACACTCTGCAACCA
Ccl4	NM_013652	GAAACAGCAGGAAGTGGGAG	CATGAAGCTCTGCGTGTCTG
Bambi	NM_026505	TGAGCAGCATCACAGTAGCA	CGCCACTCCAGCTACTTCTT
Gli1	NM_010296	GGTCTCGGGGTCTCAAACTG	CCATTCTCTGGTGGGGTTCC
Ptch1	XM_006517159	AATGTACTACGGCGTGTCCG	GACGCACAGGTAACAGGGTC
Bcl-2	NM_009741	CTT TCT GCT TTT TAT TTC ATG AGG	CAG AAG ATC ATG CCG TCC TT
Cyclin D1	NM_007631	GGGTGGGTTGGAAATGAAC	TCCTCTCCAAAATGCCAGAG
PCNA	NM_011045	AGGGTTGGTAGTTGTCGCTG	CAAACATGGTGGCGGAGTTG
PDGFRB	NM_008809	CTCCTTCAAGCTGCAGGTCA	CTCTGCAGGTAGACCAGGTG
TGFBR1	NM_009370	AGACCATCTGTCTCACAGGTAAAA	CTCCTCATCGTGTTGGTGG
TGFBR2	NM_009371	CTGGCCATGACATCACTGTT	GTCGGATGTGGAAATGGAAG
HPRT	NM_013556	GTTAAGCAGTACAGCCCCAAA	AGGGCATATCCAACAACAAACTT

### Statistical Analysis

Values are expressed as mean ± SD. Statistical differences between two groups were analyzed by the unpaired student’s t test and differences between multiple groups of data were analyzed by one-way ANOVA with Bonferroni correction (GraphPad Prism 5.0). *P* < 0.05 was considered statistically significant.

## Results and Discussion

### Attenuated hepatic fibrosis in NOX1KO and NOX4KO mice compared with WT mice after CCl4 treatment

**Fig 1:**
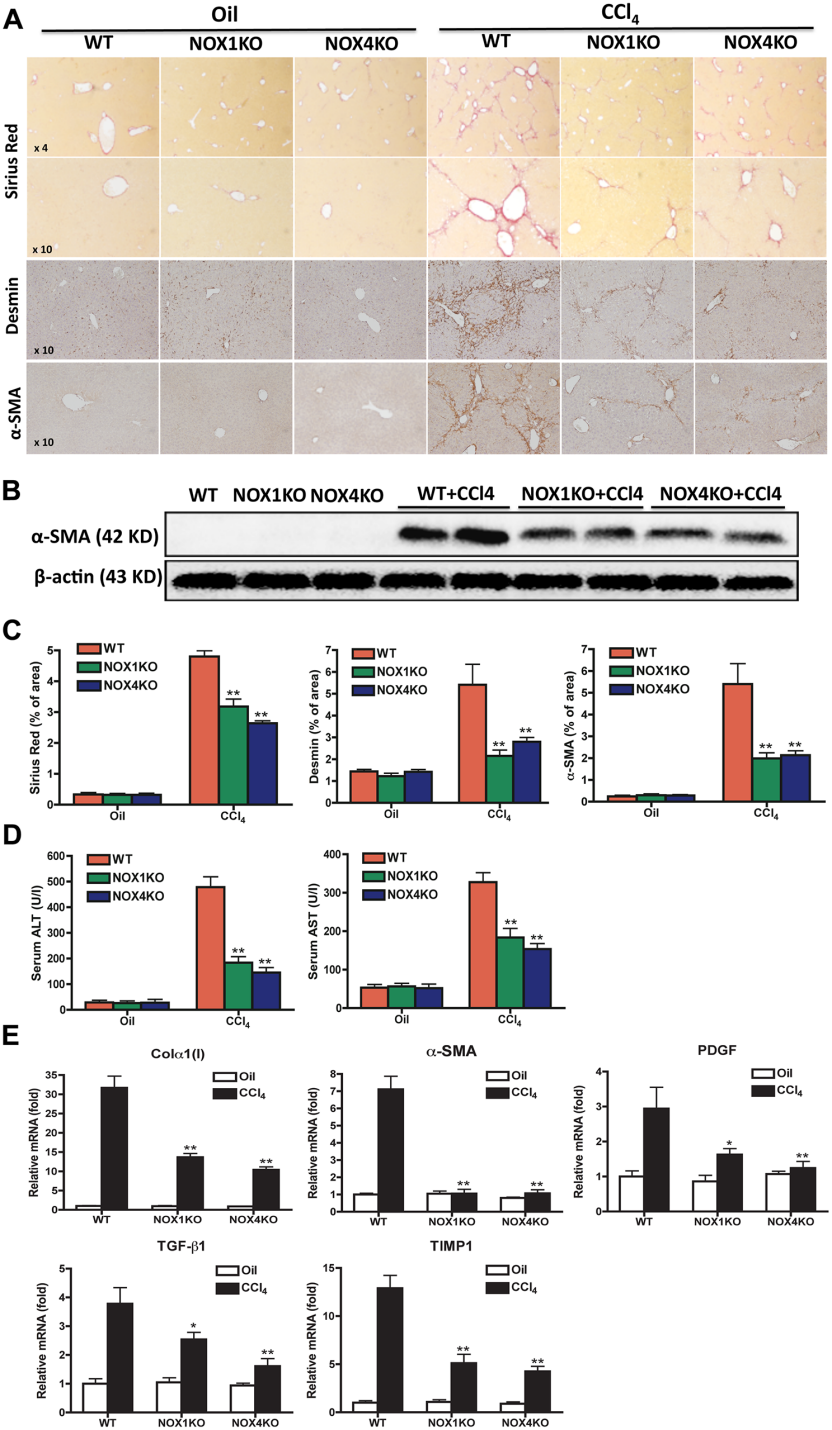
Hepatic fibrosis was attenuated in NOX1KO and NOX4KO mice after CCl_4_ injury. Livers were obtained from WT, NOX1KO, and NOX4KO mice by 12 intragastric administrations with CCl_4_ for 6 weeks, twice a week. (A) Representative images of Sirius red staining, immnohistochemistry stainings of desmin and α-SMA are shown. Original magnification X4 and X10. (B) Immunoblotting of α-SMA in liver tissues. (C) Liver function was assessed by ALT and AST. (D) Quantification of morphometric analysis of the sirius red staining and immunochemistry of desmin and α-SMA. (E) Hepatic mRNAs of fibrogenic genes were measured in WT, NOX1KO and NOX4KO mice after CCl_4_ treatment by way of quantitative real-time PCR. HPRT was used as an internal control. The data are shown as fold mRNA induction compared with control mice. *P <0.05, **P < 0.01.

To investigate the roles of NOX1 and NOX4 in hepatic fibrosis, liver fibrosis was induced by CCl_4_ in knock-out and WT mice and was assessed by morphometric analysis quantification of Sirius red staining. Hepatic fibrosis was significantly decreased in NOX1KO and NOX4KO mice compared with WT mice after CCl_4_ injections (Fig 1A and 1C). We next investigated the activation of HSCs in NOX1KO, NOX4KO and WT mice after treatment with CCl_4_. Fig 1A showed that fewer Desmin^+^α-SMA^+^ HSCs in livers of CCl_4_-treated NOX1KO and NOX4KO mice, compared to WT mice. In concordance, western blotting results showed that the expression of α-SMA was higher in fibrotic liver from WT mice than in NOX1KO and NOX4KO mice (Fig 1B).

Based on serum ALT and AST measurements, liver injury was decreased in NOX1KO and NOX4KO mice compared with WT mice in response CCl_4_ treatment (Fig 1D). In addition, the hepatic mRNA levels of fibrosis-related genes, including collagen α1(I), α-SMA, PDGF, TGF-β1, TIMP1 were significantly decreased in NOX1KO and NOX4KO mice compared with WT mice after CCl4 treatment (Fig 1E). These results demonstrate that both NOX1 and NOX4 contribute to liver fibrosis induced by CCl_4_ in mice through activation of HSCs and elevation of fibrosis-related genes expression.

### Liver inflammation was attenuated in NOX1KO and NOX4KO mice after CCl_4_ injury

**Fig 2:**
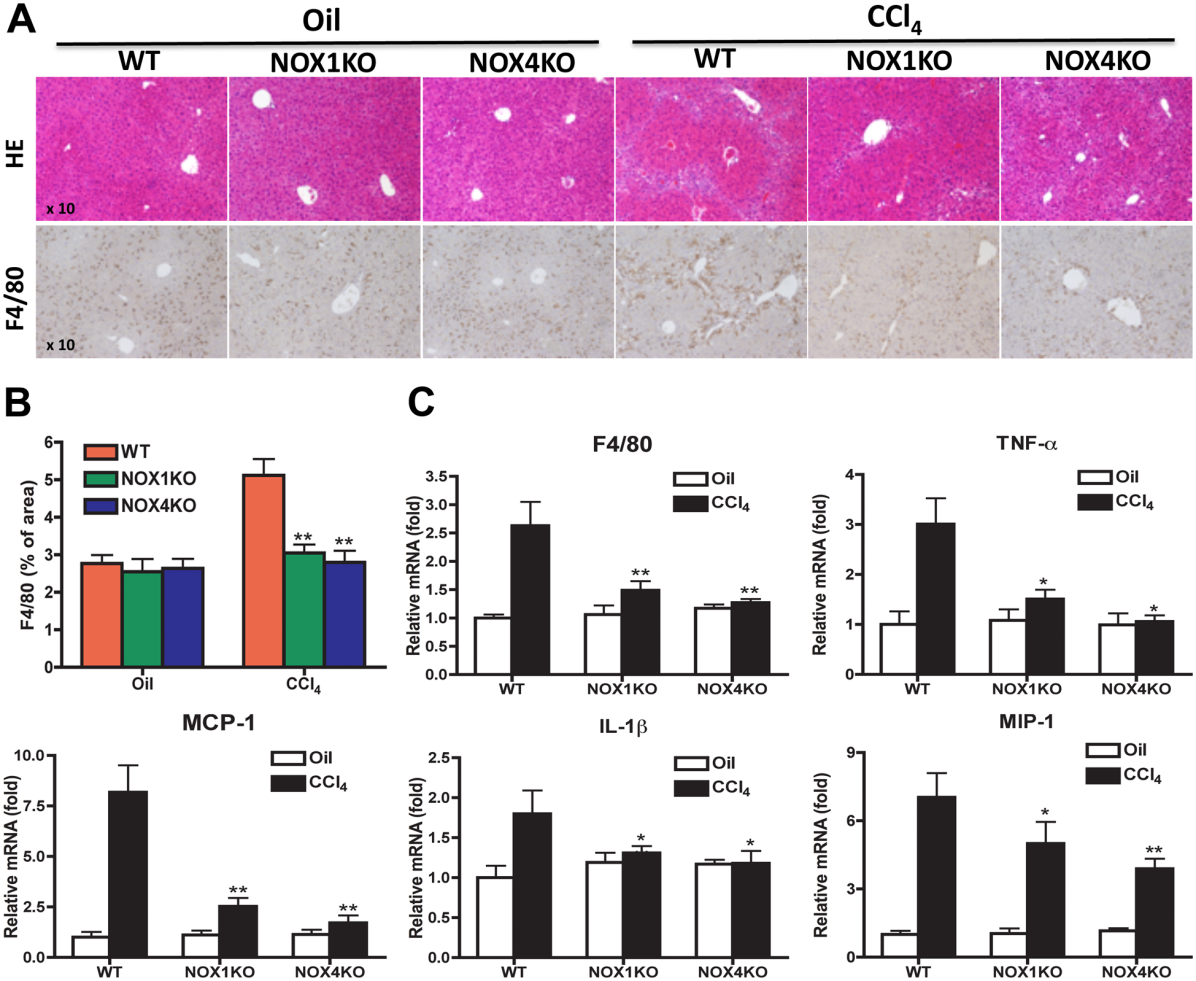
Liver injury and inflammation were attenuated in NOX1KO and NOX4KO mice after CCl_4_ injury. Livers were obtained from WT, NOX1KO, and NOX4KO mice by 12 intragastric administrations with CCl_4_ for 6 weeks, twice a week. (A) H&E staining of liver sections and F4/80 immunohistochemistry staining with (C) its quantification. Original magnification X10. (C) Hepatic mRNAs of inflammatory genes was measured in WT, NOX1KO and NOX4KO mice after CCl_4_ injury by way of quantitative real-time PCR. HPRT was used as an internal control. The data are shown as fold mRNA induction compared with control mice. *P <0.05, **P < 0.01.

To investigation the roles of NOX1 and NOX4 in liver inflammation, hepatic macrophage infiltration and inflammatory genes were evaluated. After CCl_4_ treatment, HE staining showed that inflammatory cell infiltration was decreased in NOX1KO and NOX4KO mice versus WT mice (Fig 2A). The expression of F4/80, macrophage marker, was significantly decreased in NOX1KO and NOX4KO mice compared with WT mice, as determined by IHC and quantitative real-time PCR (Fig 2A and 2B). In addition, hepatic mRNA expression of proinflammatory cytokines (TNF-α, MCP-1, IL-1β and MIP-1) was increased in WT mice after CCl_4_ treatment, but the increase was blunted in NOX1KO and NOX4KO mice (Fig 2C). These results indicate that increased liver inflammation in CCl_4_-treated WT mice was blunted in NOX1KO and NOX4KO mice.

### NOX1KO and NOX4KO mice have reduced lipid peroxidation compared with WT mice after CCl_4_ treatment

**Fig 3:**
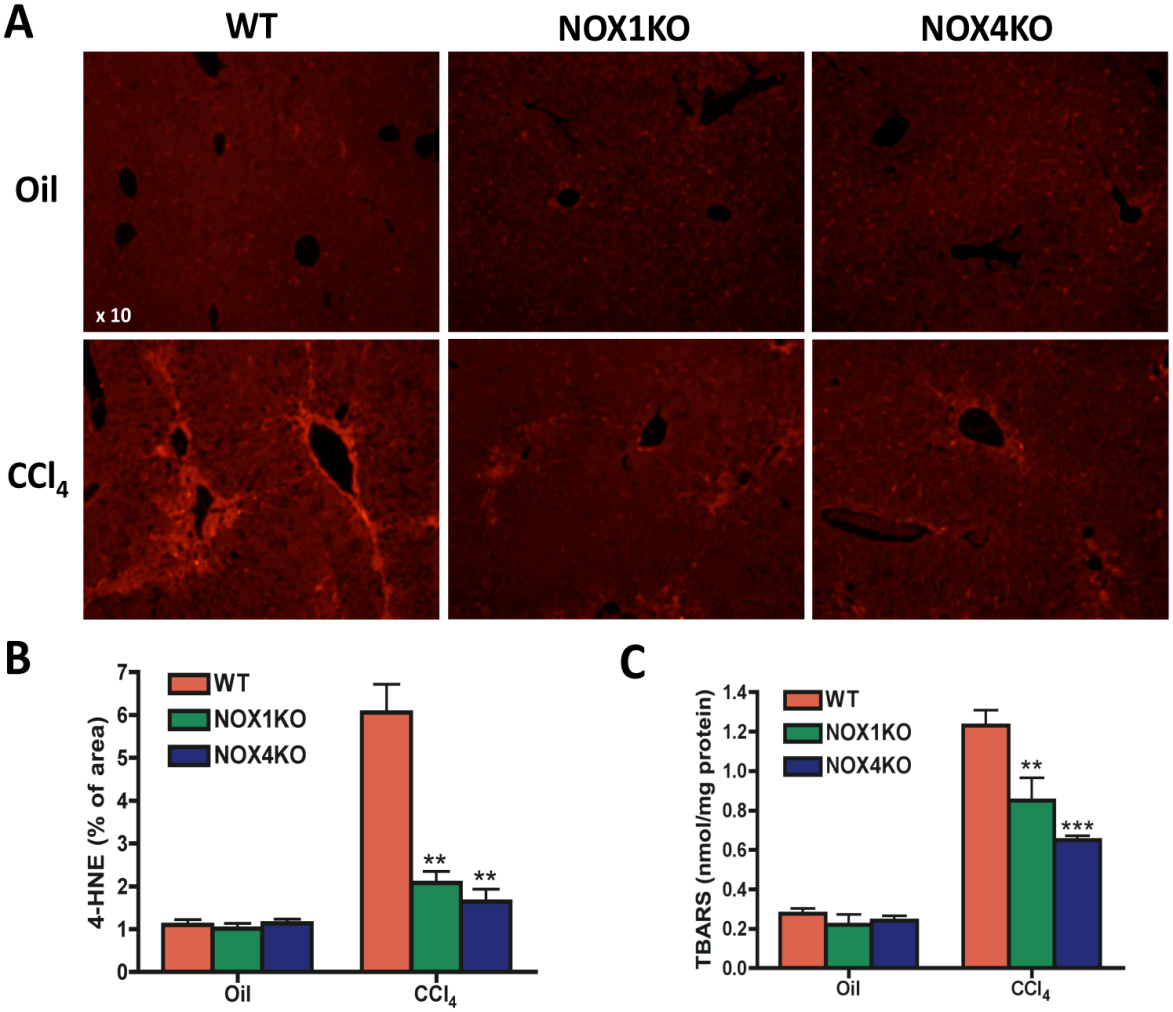
Lipid peroxidation was attenuated in NOX1KO and NOX4KO mice compared with WT mice after CCl_4_ injury. Livers were obtained from WT, NOX1KO, and NOX4KO mice by 12 intragastric administrations with CCl_4_ for 6 weeks, twice a week. (A) Representative images of 4-hydroxynonenal (4-HNE) immunoflurescent staining and its quantification (B). Original magnification X10. (C) Hepatic malondialdehyde levels were measured using thiobarbituric acid reactive substances (TBARS) assay. *P <0.05, **P < 0.01.

We measured the lipid peroxidation products 4-hydroxynonenal (4-HNE) and malondialdehyde as parameters of oxidative stress in the liver in NOX1KO, NOX4KO and WT mice after CCl_4_ treatment. Immunofluorescence staining showed that hepatic 4-HNE levels in NOX1KO and NOX4KO mice less elevated compared with WT mice after CCl_4_ treatment. Measurement of malondialdehyde showed that NOX1KO and NOX4KO mice have lower levels of lipid peroxidation compared with WT mice after CCl_4_ treatment (Fig 3), suggesting that both NOX1 and NOX4 play key roles in the formation of hepatic oxidation stress in response to CCl_4_ treatment in mice.

### NOX1 and NOX4 mediate Ang II-induced ROS production in HSCs

**Fig 4:**
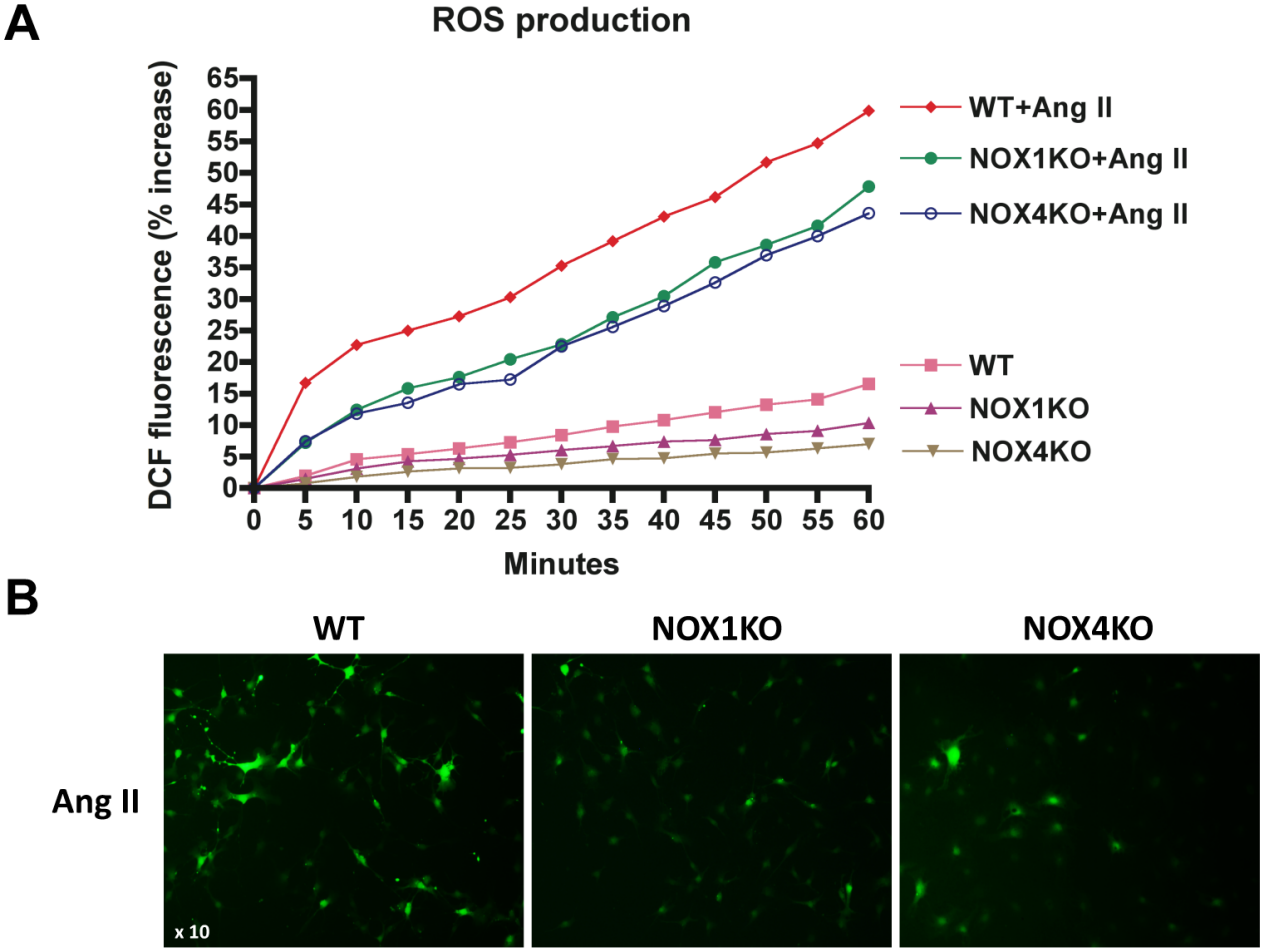
ROS production was attenuated by NOX1/4 inhibition in HSCs in response to Ang II stimulation. (A) HSCs from WT, NOX1KO and NOX4KO mice were loaded with H2DCFDA (10 μM) for 20 min. Cells were then washed and subsequently induced with Ang II (10^-6^ M). ROS production was assessed by fluorescent signals quantified continuously for 60 min using a fluorometer. (B) Representative images of ROS production in HSCs. Original magnification X10.

To identify the NOX members required for ROS generation in HSCs, we examined ROS generation in HSCs from WT, NOX1KO and NOX4KO mice. We quantitated the ROS generation in DCFDA-loaded HSCs after treatment with Ang II, a NOX agonist associated with liver fibrosis. Cells treated with buffer showed 10% increase in WT HSCs within 60 min, representing basal ROS production. In contrast, there was 4–5% increase in NOX1KO and NOX4KO HSCs (Fig 4A). Ang II induced 55%-60% increase in ROS production in WT HSCs, while there was a 40%-45% increase in NOX1KO and NOX4KO HSCs. In addition, Ang II induced higher DCFDA fluorescence in WT HSCs, but this fluorescence enhancement was suppressed in NOX1KO and NOX4KO mice (Fig 4B). These results suggest that both NOX1 and NOX4 mediate ROS production in HSCs in response to Ang II.

### GKT137831 blocks ROS production in HSCs exposed to LPS, PDGF and Shh

**Fig 5:**
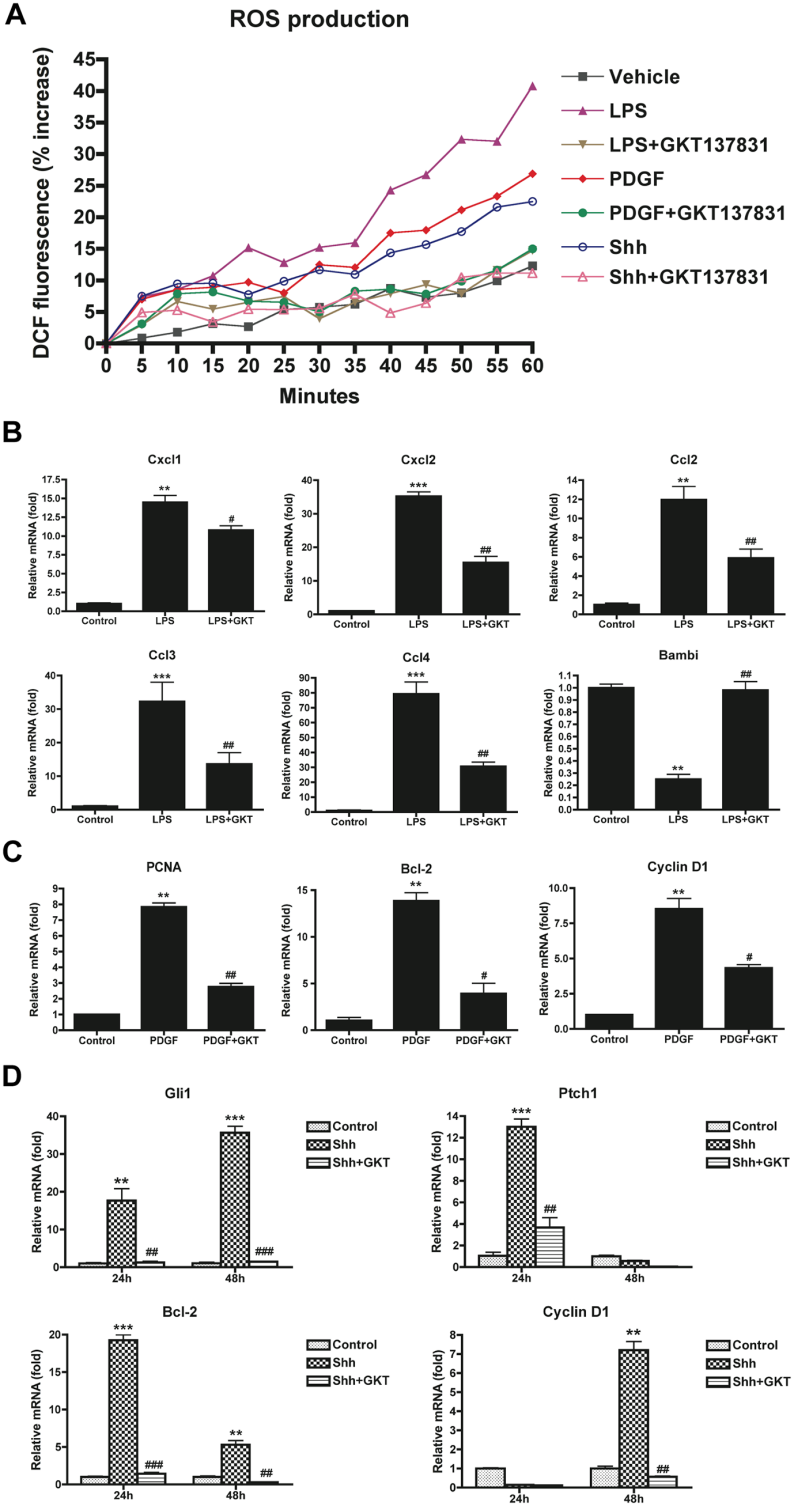
ROS production and gene expression were attenuated by GKT137831 in HSCs in response to LPS, PDGF and Shh stimulation. (A) HSCs isolated from WT mice were loaded with H2DCFDA (10 μM) for 20 min. Cells were then washed and subsequently induced with LPS (100 ng/ml), PDGF (10 ng/ml) and Shh (1 μg/ml) in the presence or absence of GKT137831 (5 μM). ROS production was assessed by fluorescent signals quantified continuously for 60 min using a fluorometer. (B) Chemokine genes were attenuated by GKT137831 in HSCs in response to LPS. HSCs isolated from WT mice were pretreated with GKT137831 (20 μM) for 30 min, and then treated with LPS (100 ng/ml) for 6 h. Chemokine genes were analyzed by quantitative real-time PCR. **P < 0.01, ***P < 0.001 vs control; #*P* < 0.05, ##*P* < 0.01 vs LPS. (C) Proliferative genes were attenuated by GKT137831 in HSCs in response to PDGF. HSCs isolated from WT mice were treated with PDGF (10 ng/ml) in the presence or absence of GKT137831 (20 μM) for 24 h. Proliferative genes were analyzed by quantitative real-time PCR. **P < 0.01 vs control; #*P* < 0.05, ##*P* < 0.01 vs PDGF. (D) Hedgehog genes were attenuated by GKT137831 in HSCs in response to hedgehog ligand Shh. HSCs isolated from WT mice were treated with Shh (1 μg/ml) in the presence or absence of GKT137831 (20 μM) for 24 and 48 h. Hedgehog genes were analyzed by quantitative real-time PCR. HPRT was used as an internal control. **P < 0.01, ***P < 0.001 vs control; ##*P* < 0.01, ###*P* < 0.001 vs Shh.

Liver fibrosis is characterized by secretion of inflammatory and fibrotic cytokines. Hedgehog (Hh) ligands, platelet derived growth factor (PDGF), and lipopolysaccharide (LPS) activate signaling pathways in HSCs to promote liver fibrosis. Therefore, we tested ROS production in HSCs treated with LPS, PDGF or the Hh ligand Shh. ROS production in HSCs is increased by LPS, PDGF and Shh (Fig 5A). GKT137831 prevented ROS generation in HSCs to the similar levels as HSCs treated with vehicle alone (Fig 5A), implying that NOX1 or NOX4 is required to generate the ROS by these ligands.

### GKT137831 attenuates chemokine upregulation in LPS-stimulated HSCs

We next examined the effects of GKT137831 on chemokines production in response to LPS in HSCs. Many chemokines (Cxcl1, Cxcl2, Ccl2, Ccl3 and Ccl4) were upregulated by LPS, and this this increase was blunted by GKT137831 treatment (Fig 5B). As previously demonstrated [18], expression of the TGF-β pseudoreceptor Bambi is strongly downregulated in HSCs after LPS stimulation (Fig 5B). GKT137831 maintains Bambi mRNA at control levels in LPS-treated HSCs, suggesting that LPS-mediated Bambi downregulation requires NOX1/4 (Fig 5B). These data demonstrated that NOX1/4 inhibition attenuated LPS-induced inflammation signaling in HSCs.

### GKT137831 attenuates PDGF-induced proliferative genes in HSCs

The mRNAs of the proliferative markers PCNA, Bcl-2 and Cyclin D1 were increased by PDGF, the most potent mitogen in HSCs. GKT137831 blocked these increases in mRNA levels (Fig 5C).

### GKT137831 blocks hedgehog pathway in HSCs

The effects of GKT137831 on the fibrogenic hedgehog pathway were also investigated. The data showed that Gli1 and its downstream genes Ptch1, Bcl-2 and Cyclin D1 were significantly increased by the hedgehog ligand Shh at 24 or 48 h. However, GKT137831 attenuates the increased mRNA levels of Gli1, Ptch1, Bcl-2 and Cyclin D1, suggesting that NOX1/4 signaling regulates the hedgehog pathway in HSCs (Fig 5D).

### NOX1 and NOX4 increase HSC proliferation

**Fig 6:**
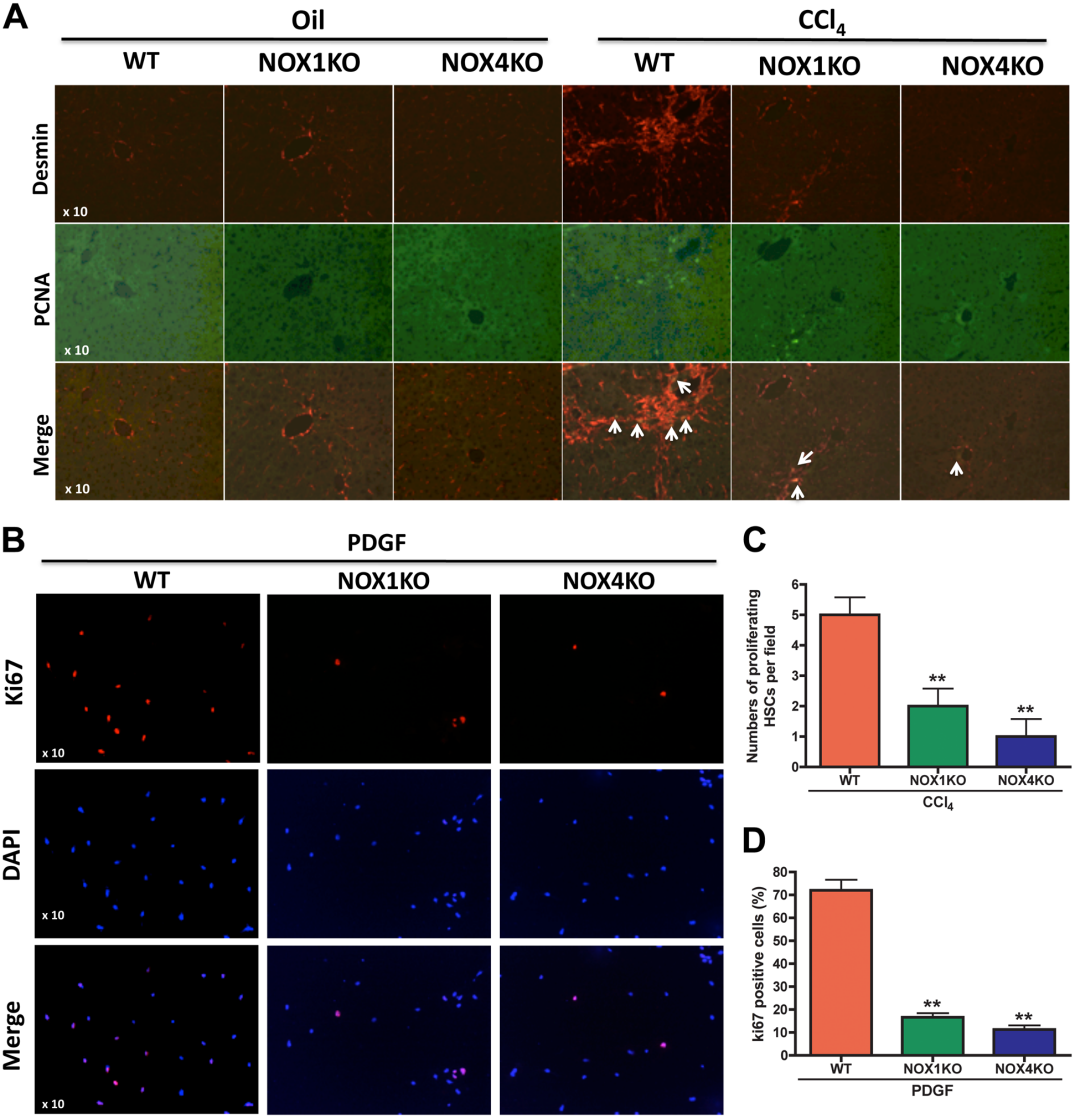
Proliferation of HSCs was reduced by deficiency of NOX1 and NOX4 in response to liver injury and PDGF. (A) HSC proliferation is reduced in NOX1KO and NOX4KO mice after CCl_4_ injury. HSC proliferation in liver was presented by PCNA immunofluorescence microscopy. Desmin is a specific marker of HSCs (Red). PCNA is a marker of proliferation (Green). Original magnification X10. (B) Proliferation was reduced in HSCs lacking NOX1 and NOX4 in response to PDGF (10 ng/ml) for 24 h. Proliferative HSCs were presented by Ki67 (Red) immunofluorescence microscopy. Nuclei were presented by DAPI (Blue). Original magnification X10. (C) Graph of PCNA-positive HSCs in vivo. (D) Graph of Ki67-positive HSCs in vitro.

We performed double immunofluorescence staining of desmin and PCNA to determine the effects of NOX1 and NOX4 on HSC proliferation in the livers of WT, NOX1KO and NOX4KO mice after CCl_4_ injury. Proliferating HSCs (desmin^+^PCNA^+^ cells) were increased in WT mice. However, there were fewer proliferated HSCs in NOX1KO and NOX4KO mice (Fig 6A and 6C).

**Fig 7:**
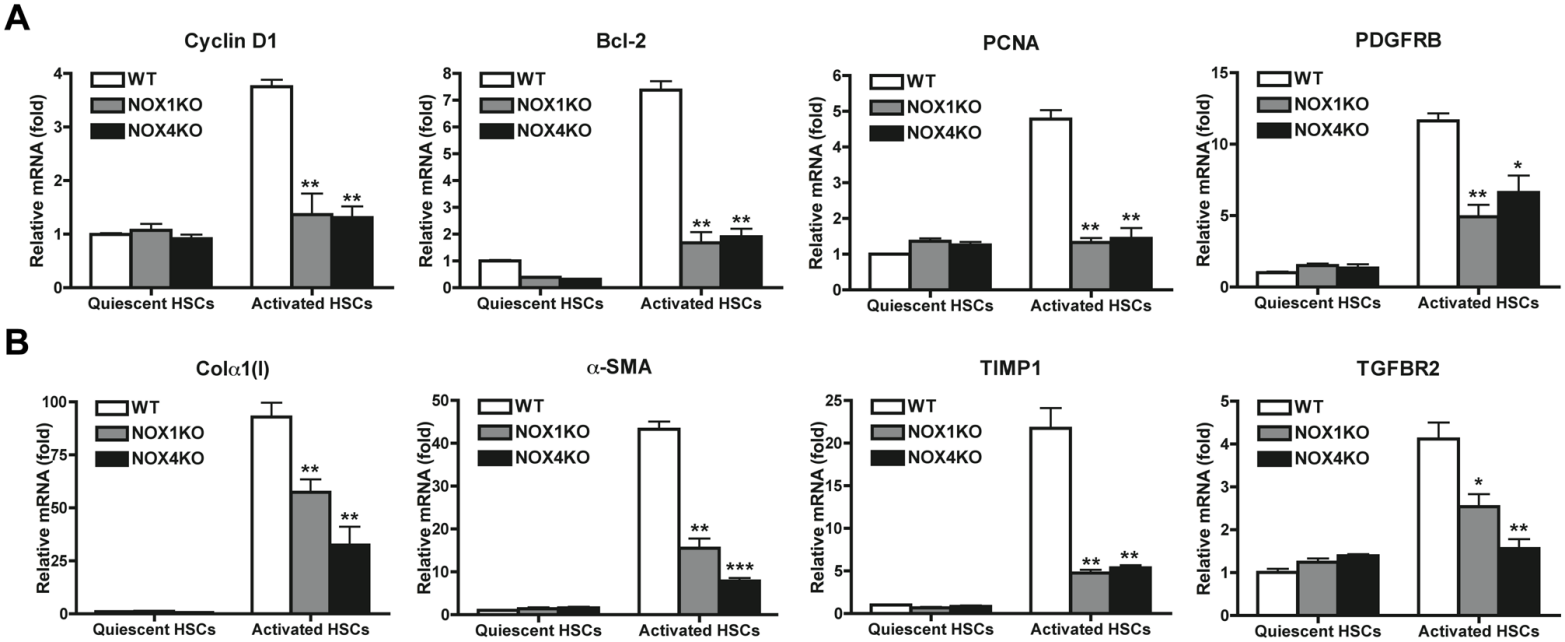
Both NOX1 and NOX4 mediate proliferative and fibrogenic responses in HSCs. HSCs from WT, NOX1KO and NOX4KO mice were cultured for 1 day (quiescent HSCs) and 5 days (activated HSCs) with 10% FBS in DMEM. mRNA expression of proliferative genes (A) and fibrogenic genes (B) were measured by quantitative real-time PCR. HPRT was used as an internal control. *P <0.05, **P < 0.01, ***P < 0.001 vs activated WT HSCs control.

We next investigated whether similar findings are observed in cultured HSCs treated with PDGF. As expected, the number of Ki67^+^HSCs isolated from WT was increased by PDGF treatment. However, PDGF produced significantly fewer Ki67^+^HSCs from NOX1KO and NOX4KO mice (Fig 6B and 6D). Furthermore, upregulation of proliferative genes (Cyclin D1, Bcl-2, PCNA and PDGFRB) was observed in activated WT HSCs (5 day plastic culture with 10% FBS medium). Proliferation of HSCs was attenuated by NOX1 and NOX4 deficiency. However, there was no change of proliferative genes in quiescent HSCs (1 day plastic culture) (Fig 7A). These results suggest that NOX1 and NOX4 mediate proliferation of HSCs in response to proliferative stimulation.

### Fibrogenesis of HSCs was attenuated by NOX1 and NOX4 deficiency

We assessed the role of NOX1 and NOX4 on fibrogenic mediators in HSCs. We measured mRNA levels of fibrogenic genes (Colα1(I), α-SMA, TIMP1 and TGFBR2) in HSCs that were isolated from WT, NOX1KO and NOX4KO mice. These fibrogenic genes were induced in activated WT HSCs (after 5 days of culture on plastic). Deficiency of NOX1 or NOX4 suppressed the induction of mRNA of pro-fibrotic genes in activated HSCs, suggesting that NOX1 and NOX4 mediate multiple fibrogenic responses in activated HSCs (Fig 7B).

### NOX1 and NOX4 are increased in patients with cirrhosis

**Fig 8:**
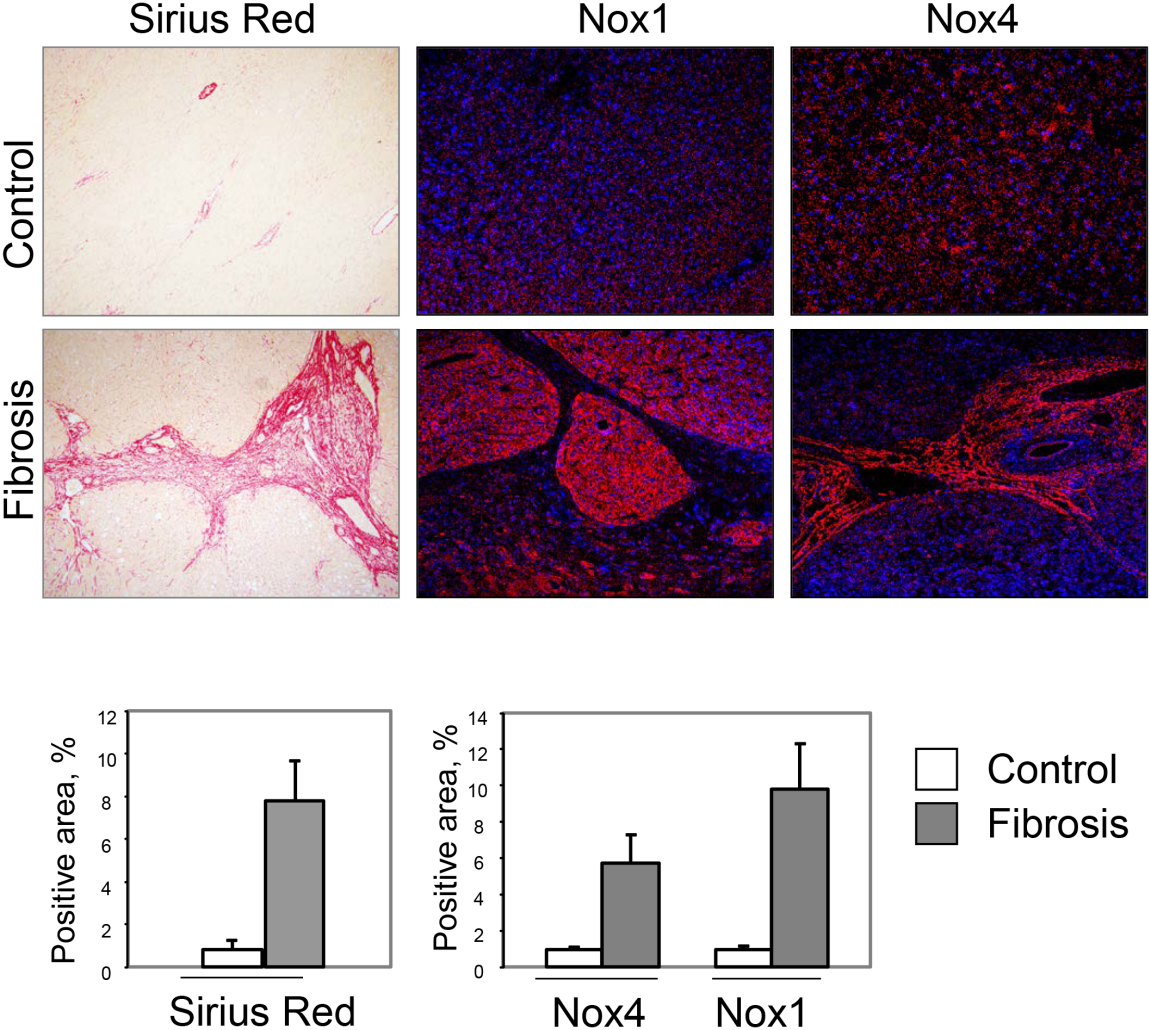
The protein levels of NOX 1 and NOX4 are increased in patients with cirrhosis. Human livers from controls (N = 7) and patients with cirrhosis (N = 10) were analyzed by Sirius Red staining and by immunofluorescence with antibodies against NOX1 and NOX4. The graphs show the percent positive area of the staining or immunofluorescence expressed as mean+S.D., p<0.05 for each comparison of control vs. fibrosis.

Previous studies have demonstrated increased mRNA levels for NOX components, in particular NOX4, in the livers of patients with HCV [19] and alcoholic liver disease [20]. However, to the best of our knowledge, the actual protein levels of the NOXs in human livers have not been assessed. Therefore, we performed immunofluorescence studies using NOX1 and NOX4 antibodies in livers of controls and patients with cirrhosis, which revealed a dramatic increase in both NOX1 and NOX4 proteins in cirrhotic livers compared to control livers (Fig 8).

## Discussion

Hepatocellular injury, followed by inflammation and activation of the innate immune system, leads to fibrosis by HSC activation and extracellular matrix (ECM) deposition [1, 21]. Our data demonstrated that NOX1 and NOX4 gene deletion inhibits multiple steps in the pathogenesis of liver fibrosis. In the chronic CCl_4_ model, where significant liver fibrosis occurs due to hepatotoxicity, NOX1KO and NOX4KO mice developed less inflammation, HSC proliferation and fibrosis than WT mice.

Seven isoforms of the NOX catalytic subunit exist (NOX1-5; Duox1 and 2). NOX isoforms NOX1, NOX2 and NOX4 are expressed in the liver [9]. The classic NOX2 induces the oxidative burst required by neutrophils to kill pathogenic bacteria. Genetic deficiency of NOX2 in patients causes chronic granulomatous disease with life threatening infections. Therefore, NOX2 is not a likely target for anti-fibrotic therapy. NOX1 and NOX4 are expressed in hepatocytes, HSCs and Kupffer cells [9]. NOX1 is catalytically activated by pro-fibrogenic agonists including Ang II, LPS, and PDGF that induce HSC activation and proliferation [9, 18] [22]. On the other hand, NOX4 is a direct TGF-responsive gene and is regulated at the level of transcription. NOX1 or NOX4 deficiency has never been reported in patients, and mice lacking either NOX1 or NOX4 have no gross phenotype. Deficiency in one NOX does not lead to a compensatory increase in other NOXs [15]. Thus, both NOX1 and NOX4 are logical targets for anti-fibrotic therapy.

Our current data showed that ROS generation was decreased in NOX1KO and NOX4KO HSCs in response to Ang II. Similar to liver injury and fibrosis, NOX1KO and NOX4KO mice showed lower hepatic lipid peroxidation after CCl_4_ treatment. These data suggest that NOX1 and NOX4 mediate oxidative stress in HSCs in response to liver injury.

GKT137831 is a small molecule inhibitor of the NOX1/NOX4 isoforms, is well tolerated in several species and is currently in clinical trials, with excellent pharmacological and safety profiles [16, 23]. Previous studies by us and others [24] demonstrated that GKT137831 attenuated liver fibrosis and ROS production as well as fibrotic genes in both the CCl4 and BDL models of liver fibrosis, suggesting that NOX1/4 may be a new therapy for liver fibrosis. However, the mechanism by which NOX1, NOX4, and GKT137831 regulate HSCs activation is not clear. Our current study showed that GKT137831 attenuated ROS production in HSCs induced by Ang II, LPS and PDGF. Furthermore, the mRNA levels of chemokine genes and proliferative genes were downregulated in HSCs by GKT137831 treatment. These data suggested that GKT137831 prevents HSC activation through inhibition of inflammatory and proliferative signaling. Among the NOX family, both NOX1 and NOX4 are expressed in HSCs and may contribute to liver fibrosis [9, 25]. Significant increase in NOX1 and NOX4 mRNA was observed in the livers as well as primary HSCs activated by serum culture [5, 25].

Our current data and other studies showed that NOX1 and NOX4 were not only expressed in HSCs, but also in hepatocytes. In addition, hepatocyte apoptosis was attenuated by NOX1 and NOX4 deficiency [5, 24]. The data is consistent with our in vivo results, in the serum ALT and AST levels were significantly decreased in NOX1KO and NOX4KO mice than those in WT with liver fibrosis. Because hepatocyte apoptosis could directly activate HSCs, and decreased apoptosis by deficiency of NOX1 and NOX4 might possibly contribute to the suppression of HSC proliferation and fibrosis.

Several lines of evidence indicated that LPS/TLR4 signaling provides a mechanistic link between inflammation and fibrosis, and plays an important role in liver fibrosis [26]. LPS stimulates HSCs to produce various chemokines and adhesion molecules to recruit Kupffer cells and/or circulating macrophages [27]. In addition, some in vitro studies have demonstrated that LPS can stimulate Kupffer cells to secrete pro-inflammatory cytokines and ROS, inducing HSCs to an active phenotype. Although Kupffer cells are considered to be the major targets of TLR ligands (LPS) in the liver, our current and previous study [18] provided several evidence that HSCs are also a primary target that drive fibrogenesis in response to LPS. The activation of TLR4 signaling by LPS enhances TGF-β signaling in HSCs [18]. Bambi is a TGF-β pseudo-receptor that lacks an intracellular kinase domain and blocks signal transduction after stimulation with ligands of the TGF-β superfamily [18, 28]. The dramatic decrease of Bambi in LPS-treated HSCs suggests that TLR4-mediated downregulation of Bambi is an integral part of HSC activation and inflammation. In the present study, GKT137831 treatment blocked both increase of mRNA levels of chemokines and decrease of Bambi. Studies on the signal transduction of LPS/TLR4 in hepatic stellate cells have demonstrated that the three MAPKs [29] and AKT [30] contribute to activation. Some evidence showed that TLR4 activation can lead to ROS signaling, and ROS may participate in signaling events downstream of TLR4 via NOX activation [31, 32]. Our data demonstrated that LPS induces ROS production and chemokines in HSCs. In contrast, GKT137831 attenuated LPS-induced ROS generation and chemokines in HSCs, suggesting that NOX1 and/or NOX4 signaling mediate LPS-induced ROS production and inflammation in HSCs. The effectiveness of other PAMPs and DAMPs to activate NOXs in HSCs still needs to be assessed.

Liver fibrosis requires activation and proliferation of HSCs. NOX1KO and NOX4KO mice contained less proliferating HSCs (PCNA^+^Desmin^+^) compared with WT mice after CCl_4_ treatment. Furthermore, cell proliferation was decreased in HSCs isolated from NOX1KO and NOX4KO mice compared with WT HSCs after PDGF treatment. Previous studies have demonstrated that PDGF signals through ERK [33], AKT [34], JNK and p38 [35] to activate hepatic stellate cells.

There is emerging evidence that hedgehog (Hh), a master developmental regulator, becomes reactivated during adult wound healing [36]. The Hh pathway is activated when Hh ligands bind to their receptor Patched on the surface of Hh-responsive cells, then promotes the nuclear translocation of the transcription factors glioblastoma Gli1, Gli2 and Gli3, which control the expression of Hh target genes [37]. Like several of the key cell types involved in liver repair, HSCs are Hh responsive [38]. Hh pathway activation promotes transition of quiescent HSCs into activated HSCs, and pathway inhibition drives activated HSCs to revert back to a quiescent phenotype [39]. Hedgehog signaling controls the fate of HSCs by regulating metabolism [37]. Dying hepatocytes produce and release Hh ligands, activating Hh signaling in neighboring Hh-responsive stromal cells including HSCs, which have been shown to become proliferative and myofibroblastic in response to Hh pathway activation [39–41]. The current study now demonstrates that Shh induces ROS in HSCs and that NOX1/4 inhibition blocks the induction of ROS and the Hh responsive genes.

In conclusion, our current study demonstrates that NOX1 and NOX4 signaling mediates hepatic fibrosis through activation of HSCs. Deficiency of NOX1 and NOX4 attenuates liver fibrosis in mice after CCl_4_ treatment. Activated HSCs and ROS generation are also attenuated in HSCs lacking NOX1 and NOX4, suggesting NOX1 and NOX4 play important roles in liver fibrosis and injury through regulating inflammation, proliferation and fibrogenesis in HSCs.

## Conclusion

Here we report for the first time a direct comparison of the long-term effects of NOX1 and NOX4 deficiency in the development and progression of liver fibrosis, by comparing liver fibrosis in CCl_4_-induced NOX1KO and NOX4KO mice and their respective wild-type (WT) littermates. Furthermore, NOX1 and NOX4 are increased in patients with cirrhosis. Our results support the concept that both NOX1 and NOX4 play important roles in liver fibrosis in HSCs, and that NOX4 has a more robust role in the activation of HSCs.
